# First-line hepatic arterial infusion chemotherapy plus lenvatinib and PD-(L)1 inhibitors versus systemic chemotherapy alone or with PD-(L)1 inhibitors in unresectable intrahepatic cholangiocarcinoma

**DOI:** 10.1007/s00432-024-05795-2

**Published:** 2024-06-18

**Authors:** Yan-Song Lin, Shuo Li, Xia Yang, Rong-Ping Guo, Yu-Hua Huang, Kun-Hao Bai, Jun Weng, Jing-Ping Yun

**Affiliations:** 1 State Key Laboratory of Oncology in South China, Guangdong Provincial Clinical Research Center for Cancer, Sun Yat-Sen University Cancer Center, Guangzhou, 510060 People’s Republic of China; 2 Department of Pathology, Sun Yat-Sen University Cancer Center, Guangzhou, 510060 People’s Republic of China; 3 Department of Liver Surgery, Sun Yat-Sen University Cancer Center, Guangzhou, 510060 People’s Republic of China; 4 Department of Endoscopy, Sun Yat-Sen University Cancer Center, Guangzhou, 510060 People’s Republic of China

**Keywords:** Hepatic arterial infusion chemotherapy, Systemic chemotherapy, PD-(L)1 inhibitors, Lenvatinib, Unresectable intrahepatic cholangiocarcinoma

## Abstract

**Purpose:**

Limited treatment options exist for unresectable intrahepatic cholangiocarcinoma (ICC), with systemic chemotherapy (SC) serving as the primary approach. This study aimed to assess the effectiveness of first-line hepatic arterial infusion chemotherapy (HAIC) in combination with lenvatinib and PD-(L)1 inhibitors (HLP) compared to SC combined with PD-(L)1 inhibitors (SCP) or SC alone in treating unresectable ICC.

**Methods:**

Patient with unresectable ICC who underwent first-line treatment with HLP, SCP or SC from January 2016 to December 2022 were retrospectively analyzed. The study evaluated and compared efficacy and safety outcomes across the three treatment groups.

**Results:**

The study comprised 42, 49, and 50 patients in the HLP, SCP, and SC groups, respectively. Median progression-free survival (PFS) times were 30.0, 10.2, and 6.5 months for HLP, SCP, and SC groups. While the SC group had a median overall survival (OS) time of 21.8 months, the HLP and SCP groups hadn’t reached median OS. The HLP group demonstrated significantly superior PFS (*p* < 0.001) and OS (*p* = 0.014) compared to the others. Moreover, the HLP group exhibited the highest objective response rate (ORR) at 50.0% and the highest disease control rate (DCR) at 88.1%, surpassing the SC group (ORR, 6.0%; DCR, 52.0%) and SCP group (ORR, 18.4%; DCR, 73.5%) (*p* < 0.05). Generally, the HLP group reported fewer grades 3–4 adverse events (AEs) compared with others.

**Conclusion:**

In contrast to systemic chemotherapy with or without PD-(L)1 inhibitors, the triple combination therapy incorporating HAIC, lenvatinib, and PD-(L)1 inhibitors showcased favorable survival benefits and manageable adverse events for unresectable ICC.

**Supplementary Information:**

The online version contains supplementary material available at 10.1007/s00432-024-05795-2.

## Introduction

Intrahepatic cholangiocarcinoma (ICC) is the second most prevalent primary hepatic malignancy, accounting for about 20% of all liver cancers, surpassed solely by hepatocellular carcinoma (HCC) (Siegel et al. 2022). Over the past four decades, the incidence of ICC has exhibited a persistent upward trend (Saha et al. 2016). Manifesting as a lethal malignancy, the 5-year overall survival (OS) for ICC remains only around 9% (Yao et al. 2016). Due to its insidious clinical nature, with patients largely asymptomatic in the early stages, merely 20%–30% of ICC cases undergo complete surgical resection (Endo et al. 2008). Those deemed unresectable are limited to locoregional treatments or systemic therapy (Moris et al. 2023).

Currently, the primary systemic chemotherapy regimens for advanced or metastatic biliary tract cancer involve cisplatin plus gemcitabine (GEMCIS), yielding a constrained efficacy with a median OS of 11.7 months (Valle et al. 2010). Another systemic chemotherapy approach for biliary tract cancer, oxaliplatin plus gemcitabine (GEMOX), has demonstrated a comparable median OS of 10.4 months in contrast to GEMCIS (Kim et al. 2019).

Immune checkpoint inhibitors (ICIs) targeting the programmed cell death 1 receptor protein (PD-1) or programmed death ligand-1 (PD-L1) have shown notable improvements in survival across various cancer types in multiple clinical trials (Pardoll 2012; Topalian et al. 2015). The TOPAZ-1 phase III trial reported a survival benefit with durvalumab in combination with gemcitabine and cisplatin in patients with advanced biliary tract cancer; however, the median OS with this combination reaches only 12.8 months (Oh et al. 2022). The KEYNOTE-966 reported that pembrolizumab in combination with gemcitabine and cisplatin achieved a OS of 12.7 months, while gemcitabine and cisplatin alone achieved a OS of 10.9 months, for patients with advanced biliary tract cancer (Kelley et al. 2023). Lenvatinib, a micromolecule tyrosine kinase inhibitor extensively utilized in advanced HCC treatment (Al-Salama et al. 2019; Zhao et al. 2020), displayed promising outcomes in a phase II study involving 14 advanced ICC patients. The combination of lenvatinib with an anti-PD-1 antibody resulted in a 21.4% objective response rate (ORR), a disease control rate (DCR) of 92.9%, and a median progression-free survival (PFS) of 5.9 months (Lin et al. 2018). Hepatic arterial infusion chemotherapy (HAIC) with oxaliplatin, fluorouracil, and leucovorin (FOLFOX) has demonstrated favorable efficacy and safety in advanced HCC, and HAIC is superior to transarterial chemoembolization (TACE) for treatment of unresectable ICC (Cai et al. 2021). However, little has known for the efficacy and safety of HAIC plus lenvatinib and ICIs for managing unresectable ICC as first-line therapy to date.

Herein, this study comprehensively compares the first-line HAIC plus lenvatinib and PD-(L)1 inhibitors against chemotherapy with or without PD-(L)1 inhibitors for patients with unresectable ICC. Comparative analyses encompass clinical outcomes and tumor responses, with a concurrent evaluation and comparison of safety profiles and adverse events (AEs).

## Materials and methods

### Case selection

In this retrospective study, individuals diagnosed with unresectable ICC and undergoing systemic chemotherapy alone, systemic chemotherapy with PD-(L)1 inhibitors or hepatic arterial infusion chemotherapy (HAIC) in combination with lenvatinib and PD-(L)1 inhibitors were consecutively enrolled at our center from January 2016 to December 2022. Written informed consent for treatment participation was obtained from all enrolled patients. All cases included in the present study satisfied the following inclusion criteria: (1) aged 18–80 years; (2) histopathologically confirmed advanced ICC; (3) diagnosed as unresectable ICC with multiple lesions, vascular invasion, local lymph node metastasis, distant metastasis, etc. by experienced hepatobiliary surgeons; (4) documented receipt of primary systemic chemotherapy (GEMCIS or GEMOX) with or without PD-(L)1 inhibitors, or HAIC plus lenvatinib and PD-(L)1 inhibitors; (5) presence of measurable lesions according to Response Evaluation Criteria in Solid Tumors (RECIST) 1.1; (6) Eastern Cooperative Oncology Group (ECOG) performance status score of 0–2. Patients were excluded based on the following exclusion criteria: (1) presence of any other malignant tumors; (2) incomplete medical follow-up data for patients; (3) Child–Pugh class C.

## Treatment procedures

Two systemic chemotherapy regimens were employed in this study. In the GEMCIS regimens, each cycle consisted of cisplatin (25 mg per square meter of body-surface area), followed by gemcitabine (1000 mg per square meter) on days 1 and 8 for every 3 weeks (Valle et al. 2010). Conversely, the GEMOX regimens involved the administration of gemcitabine (1000 mg per square meter) on days 1 and 8, and oxaliplatin (100 mg per square meter) on day 1, with each cycle lasting 3 weeks (Kim et al. 2019). PD-(L)1 inhibitors therapy entailed the administration of intravenous drugs on day 1 at 3-week intervals. HAIC procedures followed a previously established protocol (Li et al. 2023, 2021; He et al. 2019a). Femoral artery puncture was performed and a catheter was placed in the artery. After successful catheterization, angiography of superior mesenteric artery and hepatic artery were performed. The catheter was intubated to the predetermined position of hepatic artery. Then the patient with indwelling catheter was shifted to the ward. The catheter was connected to the injection pump for drugs administration in the ward. The FOLFOX regimen comprised 85 or 135 mg/m^2^ of oxaliplatin, 400 mg/m^2^ of leucovorin, and 400 mg/m^2^ of fluorouracil on the first day, along with an additional 2400 mg/m^2^ of fluorouracil administered over 46 h. The patient was bedridden during drugs administration. When drugs administration ended, the catheter was pulled out, and the patient was discharged after complete hemostasis at the puncture site. The duration between HAIC cycles ranged from 4 to 8 weeks, with patients received 2 to 8 cycles of HAIC treatment. PD-(L)1 inhibitors and lenvatinib were administered within 3 days before or after the initiation of HAIC. All the treatments maintained for 24 weeks unless disease progression or intolerability for toxic effect. The anti-tumor drugs utilized for ICC therapy have been detailed in Supplementary Table 1.

## Data collection and outcomes assessments

Clinical data were collected from the medical records of our center. Demographic and clinical characteristics encompassed age, gender, hepatitis B virus (HBV) infection status, ECOG performance status, white blood cell count (WBC), platelet count (PLT), albumin (ALB), alanine transaminase (ALT), aspartate aminotransferase (AST), total bilirubin (TBIL), C-reactive protein (CRP), liver function grade (Child–Pugh), carcinoembryonic antigen (CEA), carbohydrate antigen 19–9 (CA19-9), longest tumor diameter, tumor number, macroscopic vascular invasion, lymph node metastasis, extra-hepatic metastasis, and tumor–node–metastasis (TNM) stages. Blood tests were conducted, and tumor burdens were assessed within one week before treatment initiation. Physical examinations and laboratory evaluations were undertaken to ascertain safety both before and after treatment. Tumor response was evaluated through computed tomography (CT) and magnetic resonance imaging (MRI) three months after the initial treatment, following the RECIST 1.1 (Eisenhauer et al. 2009).

OS is defined as the time interval from the commencement of first-line treatment to cancer-related death or the last follow-up. PFS is defined as the duration from first-line treatment initiation to disease progression, intrahepatic tumor relapse, the date of death from ICC, or the last follow-up date. The follow-up deadline was December 31th, 2023, and the survival of all patients were updated to the follow-up deadline. Tumor responses were categorized as complete response (CR), partial response (PR), stable disease (SD), or progressive disease (PD). The ORR comprised the combined total of CR and PR, while the DCR encompassed CR, PR, and SD. The assessment of treatment-related AEs adhered to the National Cancer Institute Common Terminology Criteria for Adverse Events (CTCAE) version 5.0.

## Statistical analysis

Non-normally distributed variables are expressed as the medians and quartiles. The comparison of baseline characteristics between groups utilized Pearson’s chi-square test and Wilcoxon rank-sum test. Survival analysis employed the Kaplan–Meier method, with the assessment of differences in survival curves accomplished through the log-rank test. Variables demonstrating a univariate *p*-value of less than 0.05 or considered to potentially impact patient prognosis were incorporated into a multivariate Cox proportional hazards regression analysis. All analyses were conducted using SPSS 25.0 software (SPSS Inc., Chicago, IL) and GraphPad Prism (version 8.0; GraphPad, Inc.). A two-tailed *p*-value of < 0.05 was considered statistically significant.

## Results

### Baseline characteristics

**Fig. 1:**
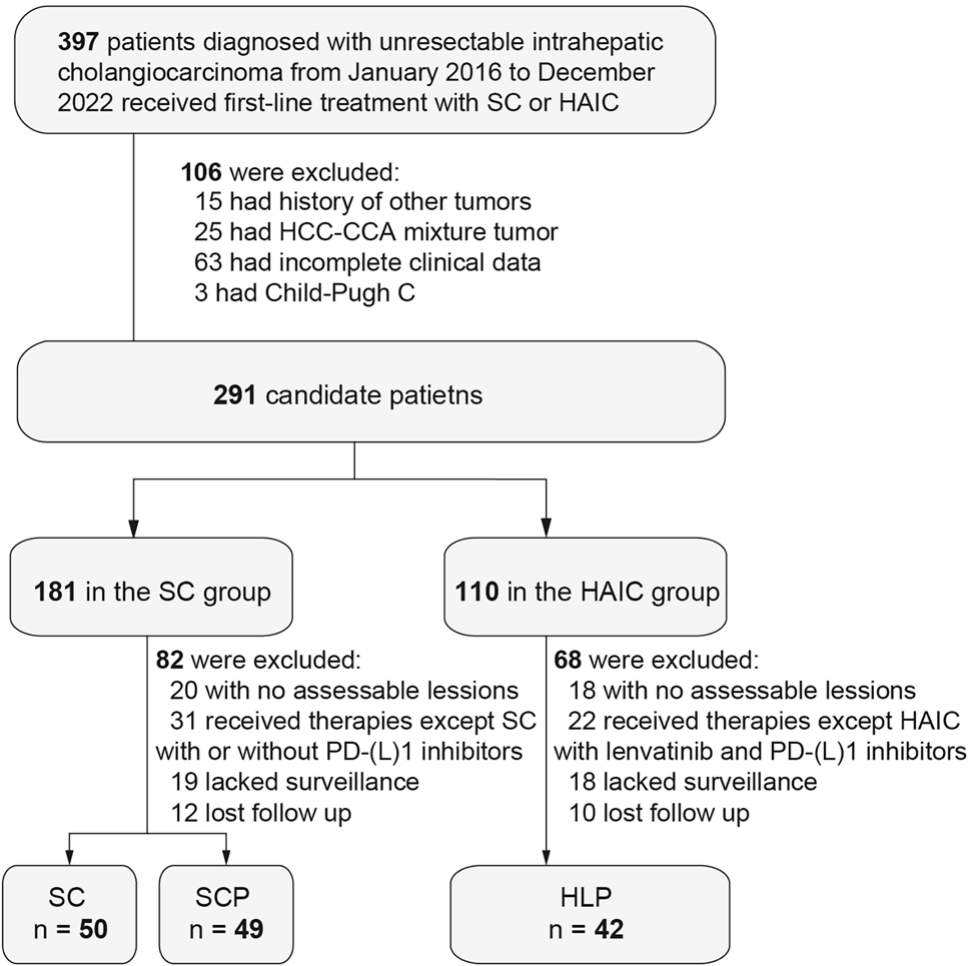
Flow diagram for patient inclusion. *SC*, systemic chemotherapy; *SCP*, systemic chemotherapy combined with PD-(L) inhibitors; *HLP*, hepatic artery infusion chemotherapy with lenvatinib and PD-(L)1 inhibitors

From January 2016 to December 2022, a comprehensive cohort comprising 141 patients diagnosed with unresectable intrahepatic cholangiocarcinoma was enrolled in this study and stratified into three distinct cohorts. Specifically, 50 patients were subjected to systemic chemotherapy (SC group), 49 patients underwent a combined treatment of systemic chemotherapy and PD-(L)1 inhibitors (SCP group), while 42 patients received HAIC concomitant with lenvatinib and PD-(L)1 inhibitors (HLP group). The procedural details pertaining to patient inclusion are delineated in Fig. 1. The baseline clinical characteristics of the subjects are summarized in Table 1. The study cohort exhibited a male predominance, constituting 83 individuals (58.9%), with a median age of 57 years (range: 48–64 years). Patients displayed the largest size of the largest nodule, measuring 6.1 cm, 6.0 cm, and 7.7 cm in the SC, SCP and HLP groups, respectively (*p* = 0.012). Conversely, other clinical parameters failed to manifest statistically significant differences across the three groups. Notably, over half of the patients presented with multiple tumors (55.3%), and a subset of 26 individuals (18.4%) exhibited macrovascular invasion. Furthermore, lymph node metastasis was observed in 90 patients (63.8%), while extra-hepatic metastasis was documented in 62 patients (44.0%). These findings collectively underscore the prevalent theme of a substantial tumor burden and advanced ICC in the confines of the present study.

**Table 1:** The baseline characteristics of study patients

Variables	SC (*n* = 50)	SCP (*n* = 49)	HLP (*n* = 42)	*p* value
Age (years)	57 (48–64)	53 (47–62)	59 (50–67)	0.216
Sex				0.540
Female	18 (36.0)	23 (46.9)	17 (40.5)	
Male	32 (64.0)	26 (53.1)	25 (59.5)	
HBV, *n* (%)				0.911
Positive	15 (30.0)	16 (32.7)	12 (28.6)	
Negative	35 (70.0)	33 (67.3)	30 (71.4)	
ECOG				0.138
0	9 (18.0)	5 (10.2)	11 (26.2)	
1–2	41 (82.0)	44 (89.8)	31 (73.8)	
WBC (10^9^/L)	7.0 (5.2–8.9)	7.2 (5.4–9.2)	8.0 (6.8–9.6)	0.177
HGB (g/L)	131 (122–144)	132 (114–142)	135 (122–148)	0.316
PLT (10^9^/L)	202 (151–265)	248 (183–295)	233 (182–281)	0.173
ALB (g/L)	41.1 (38.6–44.4)	42.5 (40.6–45.1)	43.5 (39.9–45.7)	0.067
ALT (U/L)	27.3 (14.7–42.3)	27.4 (15.1–45.7)	23.7 (18.9–39.9)	0.944
AST (U/L)	28.0 (20.5–43.1)	30.7 (21.2–51.0)	29.9 (22.5–45.1)	0.768
TBIL (μmol/L)	11.3 (8.9–15.9)	11.1 (8.7–18.5)	10.9 (8.3–16.9)	0.752
CRP (mg/L)	6.5 (1.8–18.4)	8.8 (3.9–32.3)	11.8 (2.6–39.7)	0.222
Child–Pugh				0.674
A	48 (96.0)	46 (93.9)	41 (97.6)	
B	2 (4.0)	3 (6.1)	1 (2.4)	
CEA (ng/mL)	3.8 (1.6–20.8)	2.9 (1.5–16.4)	3.8 (2.2–9.8)	0.846
CA 19–9 (U/mL)	32.6 (16.2–360.2)	75.5 (8.9–500.8)	80.4 (29.8–700.4)	0.240
Largest nodule size (cm)	6.1 (4.9–7.6)	6.0 (4.0–7.5)	7.7 (5.9–9.3)	0.012
Tumor number				0.119
Solitary	28 (56.0)	20 (40.8)	15 (35.7)	
Multiple	22 (44.0)	29 (59.2)	27 (64.3)	
TNM stage				0.052
II	7 (14.0)	5 (10.2)	12 (28.6)	
III–IV	43 (86.0)	44 (89.8)	30 (71.4)	
Tumor thrombus				0.124
Present	2 (4.0)	7 (14.3)	2 (4.8)	
Absent	48 (96.0)	42 (85.7)	40 (95.2)	
Vascular invasion				0.231
Present	9 (18.0)	6 (12.2)	11 (26.2)	
Absent	41 (82.0)	43 (87.8)	31 (73.8)	
Lymph node metastasis				0.784
Present	33 (66.0)	32 (65.3)	25 (59.5)	
Absent	17 (34.0)	17 (34.7)	17 (40.5)	
Extrahepatic metastasis				0.420
Present	19 (38.0)	25 (51.0)	18 (42.9)	
Absent	31 (62.0)	24 (49.0)	24 (57.1)	

Values are presented as median (range) or *n* (%)

*SC* systemic chemotherapy; *SCP* systemic chemotherapy with PD-(L)1 inhibitors; *HLP* hepatic artery infusion chemotherapy with lenvatinib and PD-(L)1 inhibitors; *HBV* hepatitis B virus; *ECOG* Eastern Cooperative Oncology Group; *WBC* white blood cell; *HGB* hemoglobin; *PLT* platelet count; *ALB* albumin; *ALT* alanine transaminase; *AST* aspartate transaminase; *TBIL* total bilirubin; *CRE* creatinine; *CRP* C-reactive protein; *CEA* carcinoembryonic antigen; *CA19–9*, carbohydrate antigen 19–9; *TNM* tumor–node–metastasis

### Treatment efficacy and patient survival

**Fig. 2:**
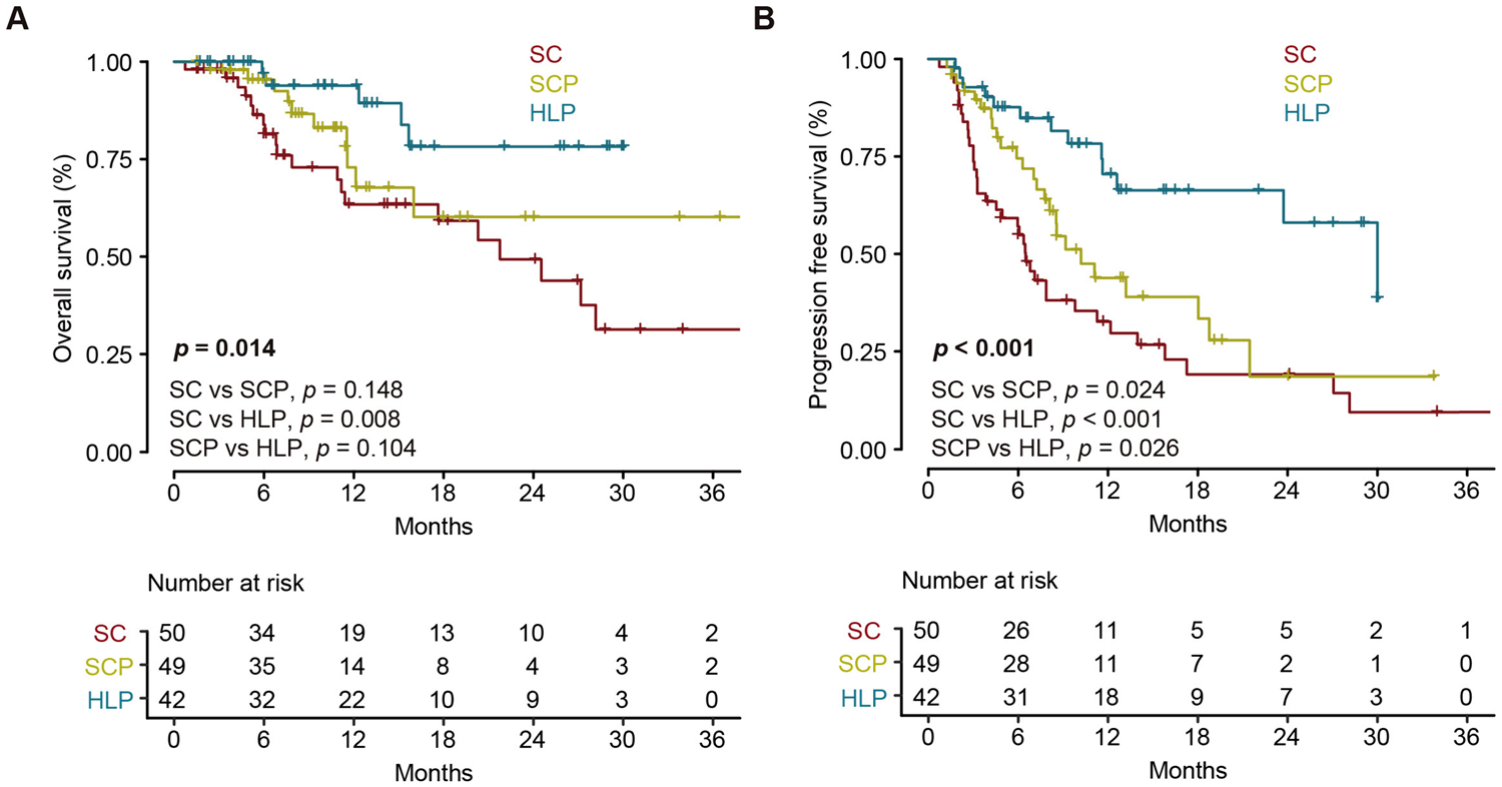
Kaplan–Meier plots for overall survival (**A)** and progression-free survival (**B**). *SC* systemic chemotherapy; *SCP* systemic chemotherapy with PD-(L)1 inhibitors; *HLP* hepatic artery infusion chemotherapy with lenvatinib and PD-(L)1 inhibitors

The median follow-up durations across the three cohorts were 15.5, 10.7, and 13.0 months, respectively. Notably, 37 patients (74.0%) in the SC group experienced disease progression, with 21 patients (42.0%) had died. In the SCP group, 25 patients (51.0%) encountered disease progression, resulting in 10 patients (20.4%) had died. In the HLP group, 13 patients (31.0%) faced disease progression, with 5 patients (11.9%) had died. The 12-month PFS were 22.0%, 22.4% and 42.9% in SC group, SCP group and HLP group, respectively The 12-month OS were 38.0%, 28.6% and 52.4% in SC group, SCP group and HLP group, respectively. The median PFS durations in the three groups were 6.5, 10.2, and 30.0 months, respectively (Table 2). In the SC group, the median OS was 21.8 months, while the SCP and HLP groups exhibited an unreached median OS. Remarkably, the HLP group demonstrated superior OS compared to the other two groups (*p* = 0.014). Upon pairwise comparisons, only the HLP group exhibited a significantly prolonged OS compared to the SC group (*p* = 0.008) (Fig. 2A). Furthermore, the HLP group showed a superior PFS compared to the other two cohorts (*p* < 0.001). In specific pairings, the SCP group demonstrated a significantly longer PFS than the SC group (*p* = 0.024), while the HLP group exhibited a significantly longer PFS than the SCP group (*p* = 0.026) (Fig. 2B). In the subgroup analysis of patients without extrahepatic metastasis, the HLP group demonstrated superior OS (*p* = 0.015) and PFS (*p* < 0.001) compared to the other two groups, while the OS and PFS in patients with extrahepatic metastasis didn’t show significant differences in three groups (Supplementary Fig. 1).

**Table 2:** Summary of tumor response between groups

Responses	SC (*n* = 50)	SCP (*n* = 49)	HLP (*n* = 42)	*p* value	Post-hoc		
					SC vs. SCP	SC vs. HLP	SCP vs. HLP
CR, *n* (%)	0 (0.0)	0 (0.0)	1 (2.4)	0.295	–	–	–
PR, *n* (%)	3 (6.0)	9 (18.4)	20 (47.6)	< 0.001	0.071	< 0.001	0.005
SD, *n* (%)	23 (46.0)	27 (55.1)	16 (38.1)	0.266	–	–	–
PD, *n* (%)	24 (48.0)	13 (26.5)	5 (11.9)	0.001	0.057	< 0.001	0.114
ORR (CR + PR), *n* (%)	3 (6.0)	9 (18.4)	21 (50.0)	< 0.001	0.071	< 0.001	0.003
DCR (CR + PR + SD), *n* (%)	26 (52.0)	36 (73.5)	37 (88.1)	0.001	0.057	< 0.001	0.114
Median follow-up, (month)	15.5	10.7	13.0	–	–	–	–
Median PFS, (month)	6.5	10.2	30.0	–	–	–	–
Median OS, (month)	21.8	Not reach	Not reach	–	–	–	–

Tumor response was evaluated at the first imaging three months after treatment, according to RECIST version 1.1

*SC* systemic chemotherapy; *SCP* systemic chemotherapy with PD-(L)1 inhibitors; *HLP* hepatic artery infusion chemotherapy with lenvatinib and PD-(L)1 inhibitors; *CR* complete response; *PR* partial response; *SD* stable disease; *PD* progression disease; *ORR* objective response rate; *DCR* disease control rate; *PFS* progression-free survival; *OS* overall survival

**Fig. 3:**
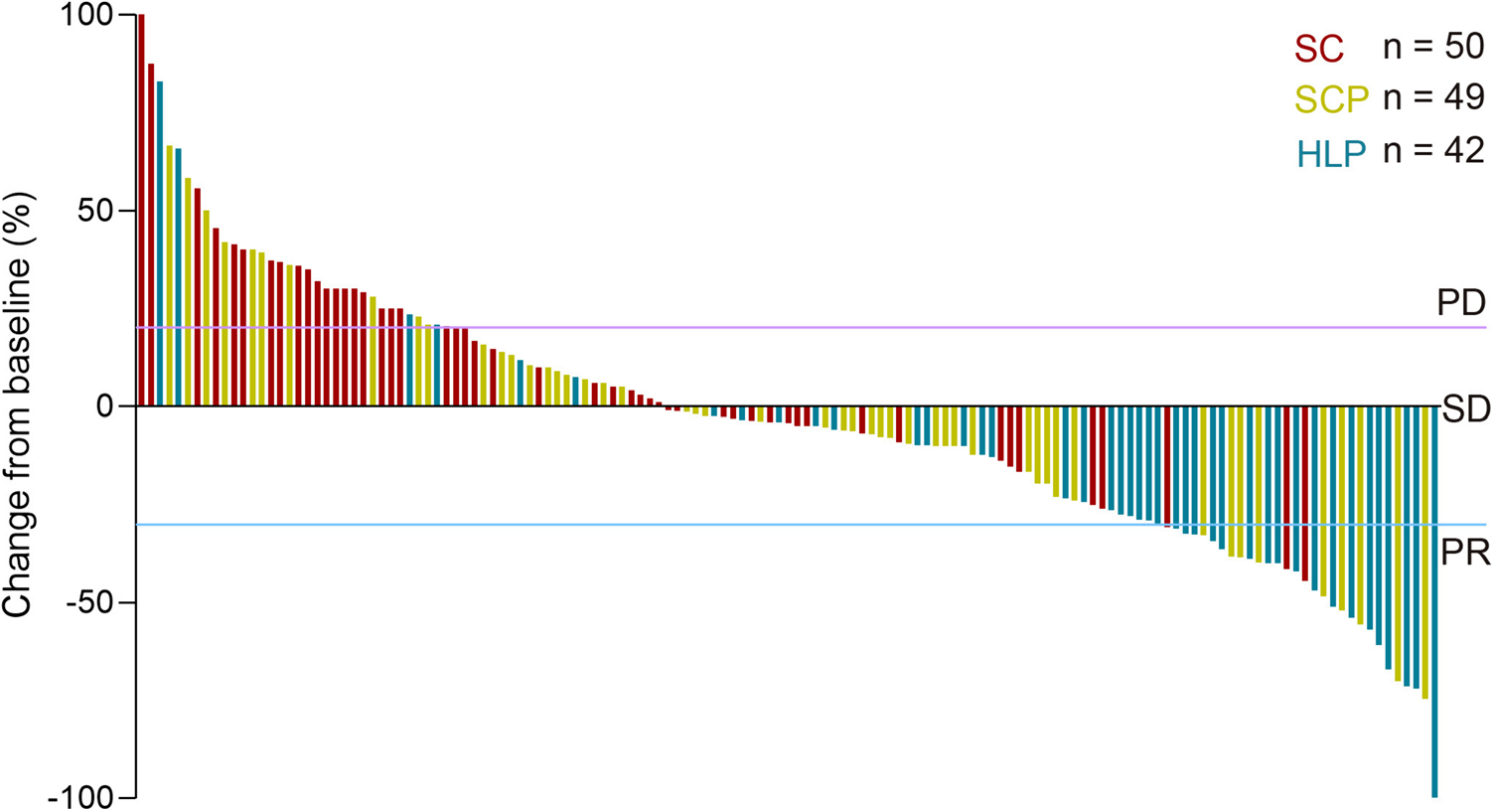
Waterfall plots for tumor size changes in intrahepatic target lesions. *SC* systemic chemotherapy; *SCP* systemic chemotherapy with PD-(L)1 inhibitors; *HLP*, hepatic artery infusion chemotherapy with lenvatinib and PD-(L)1 inhibitors; *PD* progressive disease; *SD* stable disease; *PR* partial response

Tumor response, assessed via imaging three months after treatment, according to RECIST version 1.1, revealed a CR in one patient in the HLP group, attaining the highest ORR of 50.0% and DCR of 88.1%. These figures markedly surpassed those observed in the SC group (ORR, 6.0%; DCR, 52.0%) and SCP group (ORR, 18.4%; DCR, 73.5%) (*p* all < 0.05). Additionally, the proportion of patients undergoing subsequent hepatic resection in the three groups was 0 (0.0%), 1 (2.0%), and 4 (9.5%), respectively. In inter-group analyses, the HLP group exhibited significantly higher ORR (50.0% vs. 6.0%, *p* < 0.001) and DCR (88.1% vs. 52.0%, *p* < 0.001) than the SC group. Moreover, the HLP group demonstrated a significantly higher ORR (50.0% vs. 18.4%, *p* = 0.003) than the SCP group. Although the SCP group displayed a higher ORR (18.4% vs. 6.0%, *p* = 0.071) than the SC group, this difference lacked statistical significance. In the subgroup analysis of patients without extrahepatic metastasis, HLP group attained the highest ORR of 75.0% (*p* < 0.001) and highest DCR of 100.0% (*p* < 0.001), while the ORR and DCR in patients with extrahepatic metastasis didn’t show significant differences in three groups (Supplementary Table 2–3). The optional response for intrahepatic lesions according to RECIST1.1 criteria was shown in the Fig. 3.

### Univariate and multivariate Cox regression analyses of prognostic factors

The prognostic implications of clinical variables were assessed through Cox regression analysis, with the detailed findings presented in Table 3. Univariate Cox regression analyses showed that lower levels of serum ALB, elevated CEA, an increased number of tumors, and TNM stage were significant risk factors for PFS. Concurrently, univariate Cox regression analyses for OS identified elevated CRP, increased CEA levels, and the presence of lymph node metastasis as significant risk factors. Subsequent multivariate Cox proportional analysis demonstrated that elevated CEA levels (*p* < 0.001), the number of tumors (*p* = 0.005), and TNM stage (*p* = 0.011) independently served as prognostic factors for PFS. Similarly, multivariate Cox proportional analysis for OS indicated that elevated CRP levels (*p* = 0.018), increased CEA levels (*p* = 0.023), and the existence of lymph node metastasis (*p* = 0.001) were independent prognostic factors. It is noteworthy that the inclusion of hepatic arterial infusion chemotherapy (HLP) emerged as an independent prognostic indicator for both OS (*p* = 0.009) and PFS (*p* = 0.001) when compared to SC or SCP (Table 3).

**Table 3:** COX regression analysis on the relationship between candidate prognostic factors and progression-free survival and overall survival

Variables	Progression-free survival				Overall survival			
	Univariate		Multivariate		Univariate		Multivariate	
	HR (95% CI)	*p* value	HR (95% CI)	*p* value	HR (95%CI)	*p* value	HR (95% CI)	*p* value
HLP vs. SC/SCP	0.37 (0.21–0.66)	0.001	0.35 (0.19–0.64)	0.001	0.32 (0.12–0.82)	0.017	0.28 (0.11–0.73)	0.009
Age ≥ 60	1.10 (0.68–1.78)	0.693			1.19 (0.60–2.37)	0.620		
Male	0.93 (0.58–1.49)	0.761			1.82 (0.86–3.89)	0.120		
HBV positive	0.88 (0.54–1.44)	0.603			0.78 (0.38–1.63)	0.515		
ECOG ≥ 1	1.79 (0.93–3.45)	0.081			2.44 (0.86–6.97)	0.096		
ALB ≤ 35 g/L	2.50 (1.14–5.51)	0.023			2.69 (0.81–8.95)	0.106		
TBIL > 17.1 μmol/L	1.64 (0.99–2.71)	0.053			1.75 (0.81–3.78)	0.156		
CRP > 3 mg/L	1.12 (0.68–1.87)	0.651			2.76 (1.14–6.68)	0.028	2.96 (1.21–7.25)	0.018
CEA > 5 ng/mL	1.94 (1.22–3.08)	0.005	2.38 (1.46–3.88)	< 0.001	2.06 (1.06–4.02)	0.033	2.25 (1.12–4.51)	0.023
CA 19–9 > 35 U/mL	1.55 (0.97–2.46)	0.066			1.78 (0.90–3.56)	0.100		
Chlid-Pugh: B	2.53 (0.35–18.26)	0.357			0.79 (0.11–5.80)	0.815		
Largest tumor size > 5 cm	0.97 (0.57–1.62)	0.893			1.16 (0.55–2.48)	0.696		
Tumor numbers > 1	1.67 (1.05–2.67)	0.031	2.01 (1.24–3.26)	0.005	1.65 (0.83–3.28)	0.153		
Thrombosis	1.07 (0.46–2.47)	0.881			0.33 (0.05–2.44)	0.280		
Macrovascular invasion	1.12 (0.65–1.93)	0.674			1.12 (0.51–2.47)	0.781		
Lymph node metastasis	1.53 (0.93–2.51)	0.092			3.79 (1.57–9.17)	0.003	4.94 (1.86–13.10)	0.001
Extrahepatic metastasis	1.47 (0.93–2.33)	0.096			1.74 (0.89–3.39)	0.105		
III–IV stage (TNM)	3.61 (1.56–8.35)	0.003	2.81 (1.27–6.19)	0.011	2.47 (0.87–7.03)	0.090		

*HR* hazard ratio; *CI* confidence interval; *SC* systemic chemotherapy; *SCP* systemic chemotherapy with PD-(L)1 inhibitors; *HLP* hepatic artery infusion chemotherapy with lenvatinib and PD-(L)1 inhibitors; *HBV* hepatitis B virus; *ECOG* Eastern Cooperative Oncology Group; WBC, white blood cell; *HGB* hemoglobin; *PLT* platelet count; *ALB* albumin; *ALT* alanine transaminase; AST, aspartate transaminase; *TBIL* total bilirubin; *CRE* creatinine; *CRP* C-reactive protein; *CEA*, carcinoembryonic antigen; *CA19–9* carbohydrate antigen 19–9; TNM, tumor–node–metastasis

### Adverse events and safety

AEs were comprehensively documented and are presented in Table 4. Generally, a lower incidence of AEs was observed in the HLP group. Vomiting emerged as the most prevalent AE in the SC group (20/50, 40.0%) and SCP group (20/49, 40.8%), surpassing the occurrence in the HLP group (5/42, 11.9%) (*p* = 0.004). Notably, the HLP group exhibited significantly lower occurrence rates of fatigue (14.0% [7/50] vs. 24.5% [12/49] vs. 4.8% [2/42], *p* = 0.030) and sensory neuropathy (24.0% [12/50] vs. 22.4% [11/49] vs. 4.8% [2/42], *p* = 0.033). Conversely, the incidence of hypertension was markedly higher in the HLP group (0.0% [0/50] vs. 0.0% [0/49] vs. 23.8% [10/42], *p* < 0.001). Regarding laboratory-related AEs, the HLP group exhibited significantly lower occurrence rates of leukopenia (34.0% [17/50] vs. 32.7% [16/49] vs. 11.9% [5/42], *p* = 0.032), anemia (44.0% [22/50] vs. 38.8% [19/49] vs. 19.0% [8/42], *p* = 0.033), and thrombocytopenia (36.0% [18/50] vs. 28.6% [14/49] vs. 9.5% [4/42], *p* = 0.012). Conversely, the occurrence rate of elevated AST was significantly higher in the HLP group (30.0% [15/50] vs. 26.5% [13/49] vs. 54.8% [23/42], *p* = 0.011). Furthermore, the HLP group demonstrated significantly lower occurrence rates of grades 3–4 vomiting (*p* = 0.046) and thrombocytopenia (*p* = 0.002). No significant differences were noted in other AEs between the groups, and importantly, no treatment-related deaths occurred. The immune-related adverse events of SCP and HLP groups were summarized in the Supplementary Table 4. The HLP group exhibited significantly lower occurrence rates of rash, nausea, thrombocytopenia and anemia (*p* all < 0.05), which may due to the combination with chemotherapeutic agents in SCP group.

**Table 4:** Summary of adverse events between groups

Adverse events	Any grade					Grade 3–4			
	SC (*n* = 50)	SCP (*n* = 49)	HLP (*n* = 42)	*p* value		SC (*n* = 50)	SCP (n = 49)	HLP (n = 42)	*p* value
Treatment-related AEs, *n* (%)									
Rash	11 (22.0)	11 (22.4)	2 (4.8)	0.041		0 (0.0)	0 (0.0)	0 (0.0)	1.000
Fever	7 (14.0)	7 (14.3)	6 (14.3)	0.999		0 (0.0)	0 (0.0)	0 (0.0)	1.000
Abdominal pain	16 (32.0)	12 (24.5)	6 (14.3)	0.141		2 (4.0)	0 (0.0)	0 (0.0)	0.158
Diarrhea	8 (16.0)	7 (14.3)	2 (4.8)	0.215		0 (0.0)	0 (0.0)	0 (0.0)	1.000
Vomiting	20 (40.0)	20 (40.8)	5 (11.9)	0.004		7 (14.0)	6 (12.2)	0 (0.0)	0.046
Decreased appetite	12 (24.0)	14 (28.6)	9 (21.4)	0.724		1 (2.0)	0 (0.0)	0 (0.0)	0.352
Fatigue	7 (14.0)	12 (24.5)	2 (4.8)	0.030		0 (0.0)	0 (0.0)	0 (0.0)	1.000
Sensory neuropathy	12 (24.0)	11 (22.4)	2 (4.8)	0.033		0 (0.0)	0 (0.0)	0 (0.0)	1.000
Weight loss	11 (22.0)	13 (26.5)	11 (26.2)	0.847		0 (0.0)	0 (0.0)	0 (0.0)	1.000
Hypertension	0 (0.0)	0 (0.0)	10 (23.8)	< 0.001		0 (0.0)	0 (0.0)	0 (0.0)	1.000
Laboratory-related AEs, *n* (%)									
Leukopenia	17 (34.0)	16 (32.7)	5 (11.9)	0.032		4 (8.0)	4 (8.2)	0 (0.0)	0.053
Neutropenia	14 (28.0)	12 (24.5)	5 (11.9)	0.156		5 (10.0)	4 (8.2)	1 (2.4)	0.282
Anemia	22 (44.0)	19 (38.8)	8 (19.0)	0.033		8 (16.0)	6 (12.2)	1 (2.4)	0.057
Thrombocytopenia	18 (36.0)	14 (28.6)	4 (9.5)	0.012		9 (18.0)	7 (14.3)	0 (0.0)	0.002
Hyponatremia	5 (10.0)	8 (16.3)	5 (11.9)	0.628		0 (0.0)	0 (0.0)	0 (0.0)	1.000
Hypokalemia	1 (2.0)	4 (8.2)	2 (4.8)	0.368		0 (0.0)	0 (0.0)	0 (0.0)	1.000
Elevated ALT	16 (32.0)	12 (24.5)	16 (38.1)	0.373		2 (4.0)	1 (2.0)	2 (4.8)	0.750
Elevated AST	15 (30.0)	13 (26.5)	23 (54.8)	0.011		1 (2.0)	1 (2.0)	2 (4.8)	0.692
Hypoalbuminemia	14 (28.0)	15 (30.6)	14 (33.3)	0.858		1 (2.0)	0 (0.0)	1 (2.4)	0.419
Hyperbilirubinemia	7 (14.0)	6 (12.2)	6 (14.3)	0.952		2 (4.0)	1 (2.0)	1 (2.4)	0.822
Elevated creatinine	2 (4.0)	1 (2.0)	3 (7.1)	0.483		0 (0.0)	0 (0.0)	0 (0.0)	p1.000

*AEs* adverse events; *SC* systemic chemotherapy; *SCP* systemic chemotherapy with PD-(L)1 inhibitors; *HLP* hepatic artery infusion chemotherapy with lenvatinib and PD-(L)1 inhibitors; *ALT* alanine transaminase; *AST* aspartate transaminase

## Discussion

In comparison to advanced HCC, the evolution of treatment strategies for unresectable ICC remains limited. The established primary approach for unresectable ICC continues to be chemotherapy, encompassing GEMCIS and GEMOX, which were accompanied by a series of side effects (Valle et al. 2010; Kim et al. 2019). Notably, the advent of ICI therapy in recent years has reshaped the landscape of advanced ICC treatment. Several studies indicate the effectiveness of ICI monotherapy in specific advanced biliary tract cancers (BTC). The KEYNOTE-028 study, for instance, reported a 13% ORR in advanced BTC patients treated with pembrolizumab, specifically in those with positive PD-L1 expression (Piha-Paul et al. 2020). Previous study has established that chemotherapy, including gemcitabine and 5-FU, can upregulate PD-L1 expression and modulate immune cell infiltration in cholangiocarcinoma (Koido et al. 2014). This observation suggests a potential synergistic effect when combining chemotherapy with ICI therapy. Additionally, the promising role of lenvatinib in hepatocellular carcinoma treatment has been substantiated by various clinical trials (Kudo et al. 2018; Peng et al. 2023). A phase 2 clinical trial from China, employing a first-line regimen combining toripalimab, lenvatinib, and GEMOX, demonstrated an impressive 80% ORR and 93% DCR, with median OS and PFS of 22.5 and 10.2 months, respectively (Shi et al. 2023). While HAIC has achieved notable response rates and favorable outcomes in advanced HCC, its comparative efficacy in advanced ICC remains underexplored. A study comparing HAIC with first-line SC (GEMCIS and GEMOX) for advanced ICC found that HAIC resulted in significantly longer intrahepatic PFS compared to SC, with comparable OS and PFS between the two treatments (Yang et al. 2023). The combination of HAIC plus lenvatinib plus ICI therapy for advanced ICC has not been reported.

In this retrospective study encompassing 141 patients, we evaluated the efficacy of SC (GEMCIS and GEMOX) with or without PD-(L)1 inhibitors and HAIC plus lenvatinib and PD-(L)1 inhibitors (HLP) for the treatment of unresectable ICC. Notably, patients in the SC with PD-(L)1 inhibitors group (SCP) demonstrated significantly longer PFS compared to those receiving SC alone. While a similar trend was observed in OS and ORR, the differences were not statistically significant. As of now, there is no widely accepted therapy combining chemotherapy with immunotherapy for unresectable ICC. During the 2022 annual meeting of the American Society of Clinical Oncology Gastrointestinal Cancer, the TOPAZ-1 phase III study reported interim analysis results, indicating that, compared to GEMCIS alone, the addition of duvalumab to GEMCIS reduced the risk of death by 20%. Furthermore, the combination had a longer median OS (12.8 months vs. 11.5 months) (Oh et al. 2022). Another non-randomized, multicentre, open-label phase 1 study assessing nivolumab, as monotherapy or in combination with chemotherapy, in Japanese patients with biliary tract cancer reported that the median OS and PFS in nivolumab combined with GEMCIS were 15.4 months and 4.2 months, respectively, compared to 5.2 months and 1.4 months with nivolumab alone (Ueno et al. 2019). It is noteworthy that the effect of ICI monotherapy was modest, reinforcing the predominant role of chemotherapy with immunotherapy serving as a synergistic component in combined therapy.

Remarkably, patients subjected to hepatic arterial infusion chemotherapy combined with lenvatinib and PD-(L)1 inhibitors (HLP) exhibited significantly enhanced PFS and ORR in comparison to those receiving SC with or without PD-(L)1 inhibitors. Although the OS of patients in the HLP group did not demonstrate a statistically significant superiority over those in the SCP group, it was significantly superior to the SC group. HAIC, advocated as a treatment modality for advanced HCC in Asia, has shown substantial efficacy when combined with sorafenib or PD-1 inhibitors plus lenvatinib in the context of advanced HCC (Kudo et al. 2014; Chen et al. 2020; He et al. 2019b; Mei et al. 2021). This study, for the first time globally, compared the HLP regimen with the SC and SCP regimens for unresectable ICC. The findings underscore the favorable impact of the HLP regimen, potentially attributable to the synergistic antitumor effects mediated by FOLFOX agents, PD-(L)1 inhibitors, and lenvatinib. FOLFOX agents can induce ribosome biogenesis stress and immunogenic tumor cell death (Bruno et al. 2017; Tesniere et al. 2010). Lenvatinib functioned as a multiple receptor tyrosine kinases (RTKs) inhibitor, targeting VEGFR1-3, FGFR1-4, PDGFR a, RET, and KIT (Yamamoto et al. 2014). The inhibition of VEGFR and FGFR can enhance the effect of PD-1 checkpoint blockade in HCC therapy (Deng et al. 2020). Moreover, inhibition of angiogenesis can attenuate activity of chemoresistance by normalizing tumor vessels and breaks the hypoxic microenvironment of tumors (Chung et al. 2010). It is reported that antiangiogenic therapy enhanced immunotherapy effects, including an increase in the recruiting of T cells into tumors and a decrease in immunosuppressive cytokines and T regulatory cells (Terme et al. 2013). In HCC, dual anti-PD-1/VEGFR-2 therapy significantly inhibited primary tumor growth by shifting the M1/M2 ratio of tumor-associated macrophages, increasing infiltration and activation of differentiation 8-positive (CD8 +) cytotoxic T cell, and reducing infiltration of T regulatory cell (Treg) and chemokine (C–C motif) receptor 2-positive monocyte in HCC tissue (Shigeta et al. 2020). The different underlying mechanisms of these triple-combination therapy may result in synergistic antitumor effects. Notably, HAIC, by delivering higher concentrations of chemotherapeutic agents directly to the liver, holds promise for tumor control. The consideration of such a triple-combination therapy warrants further exploration through prospective and bench-scale clinical trials.

Concerning drug safety, the HLP group exhibited a generally lower incidence of AEs compared to the SC and SCP groups, with the exception of a higher occurrence of hypertension and elevated AST. Notably, appropriate management successfully controlled these events. Gastrointestinal symptoms and hematologic toxicities were common AEs in the SC and SCP groups, encompassing abdominal pain, vomiting, decreased appetite, leukopenia, anemia, and thrombocytopenia. Vomiting, leukopenia, neutropenia, anemia, and thrombocytopenia were predominant grade 3–4 AEs across all patients, with their occurrence being notably infrequent in the HLP group. Therefore, the HLP regimen emerges as a potentially safe and well-tolerated therapeutic approach for unresectable ICC patients. Furthermore, the study identified lymph node metastasis, higher CRP and higher CEA levels as factors associated with poor OS. These prognostic risk factors should be duly considered in clinical practice.

Nevertheless, this study has several limitations. First, the retrospective and nonrandomized nature of this single-center study introduces inevitable selection biases and some imbalance of baseline characteristics, necessitating validation through prospective randomized controlled trials. Second, the limitations of factors related to the prognosis may due to the small sample of our study. Third, the diversity of PD-(L)1 inhibitors employed may influence the homogeneity of treatment procedures. Fourth, the retrospective recording of adverse events outside of a clinical trial is inevitably biased and this may underestimate the toxicity. Fifth, the relatively short follow-up duration for OS, attributed to the substantial survival rate in the HLP group at inclusion (88.1%), underscores the need for extended monitoring. Finally, subsequent treatments may be a confounder. 9.5% (4/42) patients underwent subsequent hepatic resection in HLP group, this might be a favorable factor associated with prognosis.

## Conclusions

This study has shown that, in patients with unresectable ICC, the addition of ICIs to systemic chemotherapy significantly extends PFS compared to systemic chemotherapy alone. Furthermore, when compared with systemic chemotherapy with or without ICIs, the triple combination therapy involving HAIC, lenvatinib, and ICIs demonstrates favorable survival benefits along with manageable adverse events in this cohort of patients with unresectable ICC.

## Supplementary Information

Below is the link to the electronic supplementary material.

Supplementary file1 (DOCX 365 KB)

## Data Availability

Data from the study are available on the Research Data Deposit public platform (www.researchdata.org.cn). Data sharing request should be sent to the corresponding author.

## References

[CR1] Al-Salama ZT, Syed YY, Scott LJ (2019) Lenvatinib: A Review in Hepatocellular Carcinoma. Drugs 79(6):665–674. 10.1007/s40265-019-01116-x 30993651 10.1007/s40265-019-01116-x

[CR2] Bruno PM, Liu YP, Park GY et al (2017) A subset of platinum-containing chemotherapeutic agents kills cells by inducing ribosome biogenesis stress. Nat Med 23(4):461. 10.1038/nm.4291 28263311 10.1038/nm.4291 PMC5520548

[CR3] Cai Z, He C, Zhao C, Lin X (2021) Survival Comparisons of Hepatic Arterial Infusion Chemotherapy With mFOLFOX and Transarterial Chemoembolization in Patients With Unresectable Intrahepatic Cholangiocarcinoma. Front Oncol 11:611118. 10.3389/fonc.2021.611118 33868997 10.3389/fonc.2021.611118 PMC8047640

[CR4] Chen LT, Martinelli E, Cheng AL et al (2020) Pan-Asian adapted ESMO Clinical Practice Guidelines for the management of patients with intermediate and advanced/relapsed hepatocellular carcinoma: a TOS-ESMO initiative endorsed by CSCO, ISMPO, JSMO, KSMO. MOS and SSO Annals of Oncology 31(3):334–351. 10.1016/j.annonc.2019.12.001 32067677 10.1016/j.annonc.2019.12.001

[CR5] Chung AS, Lee J, Ferrara N (2010) Targeting the tumour vasculature: insights from physiological angiogenesis. Nat Rev Cancer 10(7):505–514. 10.1038/nrc2868 20574450 10.1038/nrc2868

[CR6] Deng HJ, Kan AN, Lyu N et al (2020) Dual vascular endothelial growth factor receptor and fibroblast growth factor receptor inhibition elicits antitumor immunity and enhances programmed cell death-1 checkpoint blockade in hepatocellular carcinoma. Liver Cancer 9(3):338–357. 10.1159/000505695 32647635 10.1159/000505695 PMC7325120

[CR7] Eisenhauer EA, Therasse P, Bogaerts J et al (2009) New response evaluation criteria in solid tumours: Revised RECIST guideline (version 1.1). Eur J Cancer 45(2):228–247. 10.1016/j.ejca.2008.10.026 19097774 10.1016/j.ejca.2008.10.026

[CR8] Endo I, Gonen M, Yopp AC et al (2008) Intrahepatic cholangiocardnoma - Rising frequency, improved survival, and determinants of outcome after resection. Ann Surg 248(1):84–96. 10.1097/SLA.0b013e318176c4d3 18580211 10.1097/SLA.0b013e318176c4d3

[CR9] He MK, Li QJ, Zou RH et al (2019a) Sorafenib plus hepatic arterial infusion of oxaliplatin, fluorouracil, and leucovorin vs sorafenib alone for hepatocellular carcinoma with portal vein invasion: a randomized clinical trial. JAMA Oncol 5(7):953–960. 10.1001/jamaoncol.2019.0250 31070690 10.1001/jamaoncol.2019.0250 PMC6512278

[CR10] He MK, Li QJ, Zou RH et al (2019b) Sorafenib plus hepatic arterial infusion of oxaliplatin, fluorouracil, and leucovorin vs sorafenib alone for hepatocellular carcinoma with portal vein invasion a randomized clinical trial. JAMA Oncol 5(7):953–960. 10.1001/jamaoncol.2019.0250 31070690 10.1001/jamaoncol.2019.0250 PMC6512278

[CR11] Kelley RK, Ueno M, Yoo C et al (2023) Pembrolizumab in combination with gemcitabine and cisplatin compared with gemcitabine and cisplatin alone for patients with advanced biliary tract cancer (KEYNOTE-966): a randomised, double-blind, placebo-controlled, phase 3 trial. Lancet 401(10391):1853–1865. 10.1016/S0140-6736(23)00727-4 37075781 10.1016/S0140-6736(23)00727-4

[CR12] Kim ST, Kang JH, Lee J et al (2019) Capecitabine plus oxaliplatin versus gemcitabine plus oxaliplatin as first-line therapy for advanced biliary tract cancers: a multicenter, open-label, randomized, phase III, noninferiority trial. Annals of Oncology : Official Journal of the European Society for Medical Oncology. 30(5):788–795. 10.1093/annonc/mdz058 30785198 10.1093/annonc/mdz058

[CR13] Koido S, Kan S, Yoshida K et al (2014) Immunogenic modulation of cholangiocarcinoma cells by chemoimmunotherapy. Anticancer Res 34(11):6353–6361 25368235

[CR14] Kudo M, Matsui O, Izumi N et al (2014) JSH Consensus-based clinical practice guidelines for the management of hepatocellular carcinoma: 2014 update by the liver cancer study group of Japan. Liver Cancer 3(3–4):458–468. 10.1159/000343875 26280007 10.1159/000343875 PMC4531423

[CR15] Kudo M, Finn RS, Qin S et al (2018) Lenvatinib versus sorafenib in first-line treatment of patients with unresectable hepatocellular carcinoma: a randomised phase 3 non-inferiority trial. Lancet 391(10126):1163–1173. 10.1016/S0140-6736(18)30207-1 29433850 10.1016/S0140-6736(18)30207-1

[CR16] Li SH, Mei J, Wang QX et al (2021) Postoperative adjuvant transarterial infusion chemotherapy with FOLFOX could improve outcomes of hepatocellular carcinoma patients with microvascular invasion: a preliminary report of a phase III, randomized controlled clinical Trial (vol 27, pg 5183, 2020). Ann Surg Oncol 28(SUPPL 3):874–874. 10.1245/s10434-021-09813-2 32418078 10.1245/s10434-020-08601-8

[CR17] Li SH, Mei J, Cheng Y et al (2023) Postoperative adjuvant hepatic arterial infusion chemotherapy with FOLFOX in hepatocellular carcinoma with microvascular invasion: a multicenter, phase III. Randomized Study. J Clin Oncol. 41(10):1898–1908. 10.1200/JCO.22.01142 36525610 10.1200/JCO.22.01142 PMC10082249

[CR18] Lin JZ, Shi WW, Zhao SH et al (2018) Lenvatinib plus checkpoint inhibitors in patients (pts) with advanced intrahepatic cholangiocarcinoma (ICC): Preliminary data and correlation with next-generation sequencing. J Clin Oncol 36(4):500. 10.1200/JCO.2018.36.4_suppl.500

[CR19] Mei J, Tang YH, Wei W et al (2021) Hepatic Arterial infusion chemotherapy combined With PD-1 inhibitors plus lenvatinib *versus* PD-1 inhibitors plus lenvatinib for advanced hepatocellular carcinoma. Front Oncol 11(618206):11. 10.3389/fonc.2021.618206 10.3389/fonc.2021.618206 PMC7947809 33718175

[CR20] Moris D, Palta M, Kim C, Allen PJ, Morse MA, Lidsky ME (2023) Advances in the treatment of intrahepatic cholangiocarcinoma: An overview of the current and future therapeutic landscape for clinicians. CA Cancer J Clin 73(2):198–222. 10.3322/caac.21759 36260350 10.3322/caac.21759

[CR21] Oh D-Y, He A-R, Qin S et al (2022) A phase 3 randomized, double-blind, placebo-controlled study of durvalumab in combination with gemcitabine plus cisplatin (GemCis) in patients (pts) with advanced biliary tract cancer (BTC): TOPAZ-1. J Clin Oncol 1(8):378–378. 10.1056/EVIDoa2200015

[CR22] Pardoll DM (2012) The blockade of immune checkpoints in cancer immunotherapy. Nat Rev Cancer 12(4):252–264. 10.1038/nrc3239 22437870 10.1038/nrc3239 PMC4856023

[CR23] Peng ZW, Fan WZ, Zhu BW et al (2023) Lenvatinib combined with transarterial chemoembolization as first-line treatment for advanced hepatocellular carcinoma: a phase III, randomized clinical trial (LAUNCH). J Clin Oncol 41(1):117–119. 10.1200/JCO.22.00392 35921605 10.1200/JCO.22.00392

[CR24] Piha-Paul SA, Oh DY, Ueno M et al (2020) Efficacy and safety of pembrolizumab for the treatment of advanced biliary cancer: Results from the KEYNOTE-158 and KEYNOTE-028 studies. Int J Cancer 147(8):2190–2198. 10.1002/ijc.33013 32359091 10.1002/ijc.33013

[CR25] Saha SK, Zhu AX, Fuchs CS, Brooks GA (2016) Forty-year trends in cholangiocarcinoma incidence in the US: intrahepatic disease on the rise. Oncologist 21(5):594–599. 10.1634/theoncologist.2015-0446 27000463 10.1634/theoncologist.2015-0446 PMC4861366

[CR26] Shi G, Huang X, Wu D et al (2023) Toripalimab combined with lenvatinib and GEMOX is a promising regimen as first-line treatment for advanced intrahepatic cholangiocarcinoma: a single-center, single-arm, phase 2 study. Signal Transduct Target Ther 8(4):1983–1992 10.1038/s41392-023-01317-7 PMC10020443 36928584

[CR27] Shigeta K, Datta M, Hato T et al (2020) Dual programmed death receptor-1 and vascular endothelial growth factor receptor-2 blockade promotes vascular normalization and enhances antitumor immune responses in hepatocellular carcinoma. Hepatology 71(4):1247–1261. 10.1002/hep.30889 31378984 10.1002/hep.30889 PMC7000304

[CR28] Siegel RL, Miller KD, Fuchs HE, Jemal A (2022) Cancer statistics, 2022. Ca-a Cancer Journal for Clinicians 72(1):7–33. 10.3322/caac.21708 35020204 10.3322/caac.21708

[CR29] Terme M, Pernot S, Marcheteau E et al (2013) VEGFA-VEGFR pathway blockade inhibits tumor-induced regulatory T-cell proliferation in colorectal cancer. Cancer Res 73(2):539–549. 10.1158/0008-5472.CAN-12-2325 23108136 10.1158/0008-5472.CAN-12-2325

[CR30] Tesniere A, Schlemmer F, Boige V et al (2010) Immunogenic death of colon cancer cells treated with oxaliplatin. Oncogene 29(4):482–491. 10.1038/onc.2009.356 19881547 10.1038/onc.2009.356

[CR31] Topalian SL, Drake CG, Pardoll DM (2015) Immune Checkpoint Blockade: A Common Denominator Approach to Cancer Therapy. Cancer Cell 27(4):450–461. 10.1016/j.ccell.2015.03.001 25858804 10.1016/j.ccell.2015.03.001 PMC4400238

[CR32] Ueno M, Ikeda M, Morizane C et al (2019) Nivolumab alone or in combination with cisplatin plus gemcitabine in Japanese patients with unresectable or recurrent biliary tract cancer: a non-randomised, multicentre, open-label, phase 1 study. Lancet Gastroenterology & Hepatology 4(8):611–621. 10.1016/S2468-1253(19)30086-X 31109808 10.1016/S2468-1253(19)30086-X

[CR33] Valle J, Wasan H, Palmer DH et al (2010) Cisplatin plus Gemcitabine versus Gemcitabine for Biliary Tract Cancer. N Engl J Med 362(14):1273–1281. 10.1056/NEJMoa0908721 20375404 10.1056/NEJMoa0908721

[CR34] Yamamoto Y, Matsui J, Matsushima T et al (2014) Lenvatinib, an angiogenesis inhibitor targeting VEGFR/FGFR, shows broad antitumor activity in human tumor xenograft models associated with microvessel density and pericyte coverage. Vascular Cell 2014(6):18. 10.1186/2045-824X-6-18 10.1186/2045-824X-6-18 PMC4156793 25197551

[CR35] Yang Z, Fu Y, Wu W et al (2023) Comparison of hepatic arterial infusion chemotherapy with mFOLFOX vs. first-line systemic chemotherapy in patients with unresectable intrahepatic cholangiocarcinoma. Front Pharmacol 5(14):1234342. 10.3389/fphar.2023.1234342 10.3389/fphar.2023.1234342 PMC10508288 37731737

[CR36] Yao KJ, Jabbour S, Parekh N, Lin Y, Moss RA (2016) Increasing mortality in the United States from cholangiocarcinoma: an analysis of the National Center for Health Statistics Database. BMC Gastroenterol 16:117. 10.1186/s12876-016-0527-z 27655244 10.1186/s12876-016-0527-z PMC5031355

[CR37] Zhao Y, Zhang YN, Wang KT, Chen L (2020) Lenvatinib for hepatocellular carcinoma: From preclinical mechanisms to anti-cancer therapy. Biochim Biophys Acta Rev Cancer 1874(1):188391. 10.1016/j.bbcan.2020.188391 32659252 10.1016/j.bbcan.2020.188391

