# Supplementary material for: First-line hepatic arterial infusion chemotherapy plus lenvatinib and PD-(L)1 inhibitors versus systemic chemotherapy alone or with PD-(L)1 inhibitors in unresectable intrahepatic cholangiocarcinoma
Source: J Cancer Res Clin Oncol. 2024 Jun 18;150(6):309. doi: 10.1007/s00432-024-05795-2 (PMC11189327; doi:10.1007/s00432-024-05795-2)
Supplement: Supplementary file 1 — Supplementary file1 (DOCX 365 KB) [file 432_2024_5795_MOESM1_ESM.docx]

Supplementary table 1. Summary of anti-tumor drugs for unresectable intrahepatic cholangiocarcinoma.

| **Treatment** | **Drug name** | **Administration** | **Dose** | **Frequency** |
| --- | --- | --- | --- | --- |
| Systemic chemotherapy | Gemcitabine | Intravenous | 1000mg/m^2^ | Day 1 and 8, Q3W |
|  | Cisplatin | Intravenous | 25 mg/m^2^ | Day 1, Q3W |
|  | Oxaliplatin | Intravenous | 85 mg/m^2^ | Day 1, Q3W |
| FOLFOX-HAIC | Oxaliplatin | Hepatic artery | 130 mg/m^2^ | Day 1, Q3W |
|  | Leucovorin | Hepatic artery | 400 mg/m^2^ | Day 1, Q3W |
|  | Fluorouracil | Hepatic artery | 400 mg/m^2^ day 1; 2400 mg/m^2^ over 46 h | Q3W |
| Anti-PD-(L)1 therapy | Toripalimab | Intravenous | 240 mg | Q3W |
|  | Sintilimab | Intravenous | 200 mg | Q3W |
|  | Tislelizumab | Intravenous | 200 mg | Q3W |
|  | Pembrolizumab | Intravenous | 200 mg | Q3W |
|  | Camrelizumab | Intravenous | 3mg/kg | Q3W |
|  | Nivolumab | Intravenous | 240 mg | Q2W |
|  | Durvalumab | Intravenous | 1500 mg | Q3W |
| Targeted therapy | Lenvatinib | Oral | 8 mg | QD |

Note: GECIS scheme, Gemcitabine+ Cisplatin; GEMOX scheme, Gemcitabine+ Oxaliplatin; FOLFOX-HAIC, hepatic arterial infusion chemotherapy with oxaliplatin, leucovorin and fluorouracil.

Supplementary table **2. Summary of tumor response between groups in patients without extrahepatic metastasis.**

| **Responses** | **SC (n=31)** | **SCP (n=24)** | **HLP (n=24)** | ***p* value** | **Post-hoc** | | |
| --- | --- | --- | --- | --- | --- | --- | --- |
|  |  |  |  |  | **SC vs. SCP** | **SC vs. HLP** | **SCP vs. HLP** |
| CR, n (%) | 0 (0.0) | 0 (0.0) | 1 (2.4) | 0.299 | - | - | - |
| PR, n (%) | 3 (9.7) | 7 (29.2) | 17 (70.8) | < 0.001 | 0.084 | < 0.001 | 0.009 |
| SD, n (%) | 14 (45.2) | 12 (50.0) | 6 (25.0) | 0.168 | - | - | -- |
| PD, n (%) | 14 (45.2) | 5 (20.8) | 0 (0.0) | < 0.001 | 0.087 | < 0.001 | 0.075 |
| ORR (CR+PR), n (%) | 3 (9.7) | 7 (29.2) | 18 (75.0) | < 0.001 | 0.084 | < 0.001 | 0.003 |
| DCR (CR+PR+SD), n (%) | 17 (54.8) | 19 (79.2) | 24 (100.0) | < 0.001 | 0.087 | < 0.001 | 0.075 |
| Median follow-up, (month) | 15.5 | 8.4 | 14.8 | - | - | - | - |
| Median PFS, (month) | 7.1 | 11.1 | 30.0 | - | - | - | - |
| Median OS, (month) | 24.5 | 16.0 | Not reach | - | - | - | - |

Supplementary table **3. Summary of tumor response between groups in patients with extrahepatic metastasis.**

| **Responses** | **SC (n=19)** | **SCP (n=25)** | **HLP (n=18)** | ***p* value** | **Post-hoc** | | |
| --- | --- | --- | --- | --- | --- | --- | --- |
|  |  |  |  |  | **SC vs. SCP** | **SC vs. HLP** | **SCP vs. HLP** |
| CR, n (%) | 0 (0.0) | 0 (0.0) | 0 (0.0) | - | - | - | - |
| PR, n (%) | 0 (0.0) | 2 (8.0) | 3 (16.7) | 0.100 | - | - | - |
| SD, n (%) | 9 (47.4) | 15 (60.0) | 10 (55.6) | 0.704 | - | - | - |
| PD, n (%) | 10 (52.6) | 8 (32.0) | 5 (27.8) | 0.233 | - | - | - |
| ORR (CR+PR), n (%) | 0 (0.0) | 2 (8.0) | 3 (16.7) | 0.100 | - | - | - |
| DCR (CR+PR+SD), n (%) | 9 (47.4) | 17 (68.0) | 13 (72.2) | 0.233 | - | - | - |
| Median follow-up, (month) | 7.5 | 11.3 | 9.7 | - | - | - | - |
| Median PFS, (month) | 6.0 | 9.2 | 11.6 | - | - | - | - |
| Median OS, (month) | 21.8 | Not reach | Not reach | - | - | - | - |

**Note:** Tumor response was evaluated at the first imaging three months after treatment, according to RECIST version 1.1.

Supplementary table **4. Summary of immune-related adverse events in SCP and HLP groups.**

| **Adverse events,**  **n (%)** | **Any grade** | | | |  | | **Grade 3-4** | | |
| --- | --- | --- | --- | --- | --- | --- | --- | --- | --- |
|  | **SCP**  **(n=49)** | **HLP (n=42)** | ***p* value** |  | | **SCP**  **(n=49)** | | **HLP (n=42)** | ***p***  **value** |
| Rash | 11 (22.4) | 2 (4.8) | 0.016 |  | | 0 (0.0) | | 0 (0.0) | 1.000 |
| Nausea | 20 (40.8) | 5 (11.9) | 0.002 |  | | 6 (12.2) | | 0 (0.0) | 0.055 |
| Diarrhea | 7 (14.3) | 2 (4.8) | 0.129 |  | | 0 (0.0) | | 0 (0.0) | 1.000 |
| Allergic reaction | 1 (2.0) | 1 (2.4) | 1.000 |  | | 0 (0.0) | | 0 (0.0) | 1.000 |
| Dyspnea | 0 (0.0) | 0 (0.0) | 1.000 |  | | 0 (0.0) | | 0 (0.0) | 1.000 |
| Thrombocytopenia | 14 (28.6) | 4 (9.5) | 0.023 |  | | 7 (14.3) | | 0 (0.0) | 0.031 |
| Anemia | 19 (38.8) | 8 (19.0) | 0.040 |  | | 6 (12.2) | | 1 (2.4) | 0.172 |
| Mucositis | 2 (4.1) | 1 (2.4) | 1.000 |  | | 0 (0.0) | | 0 (0.0) | 1.000 |
| Hyperthyroidism | 1 (2.0) | 1 (2.4) | 1.000 |  | | 0 (0.0) | | 0 (0.0) | 1.000 |
| Hepatitis | 12 (24.5) | 16 (38.1) | 0.161 |  | | 1 (2.0) | | 2 (4.8) | 0.892 |
| Pneumonitis | 1 (2.0) | 1 (2.4) | 1.000 |  | | 0 (0.0) | | 0 (0.0) | 1.000 |
| Arthritis | 1 (2.0) | 0 (0.0) | 1.000 |  | | 0 (0.0) | | 0 (0.0) | 1.000 |
| Nephritis | 1 (2.0) | 3 (7.1) | 0.502 |  | | 0 (0.0) | | 0 (0.0) | 1.000 |
| Pruritus | 1 (2.0) | 1 (2.4) | 1.000 |  | | 0 (0.0) | | 0 (0.0) | 1.000 |
| Myocarditis | 0 (0.0) | 0 (0.0) | 1.000 |  | | 0 (0.0) | | 0 (0.0) | 1.000 |
| Enteritis | 1 (2.0) | 1 (2.4) | 1.000 |  | | 0 (0.0) | | 0 (0.0) | 1.000 |
| Uveitis | 0 (0.0) | 0 (0.0) | 1.000 |  | | 0 (0.0) | | 0 (0.0) | 1.000 |

**Abbreviations:** SCP, systemic chemotherapy with PD-(L)1 inhibitors; HLP, hepatic artery infusion chemotherapy with lenvatinib and PD-(L)1 inhibitors.


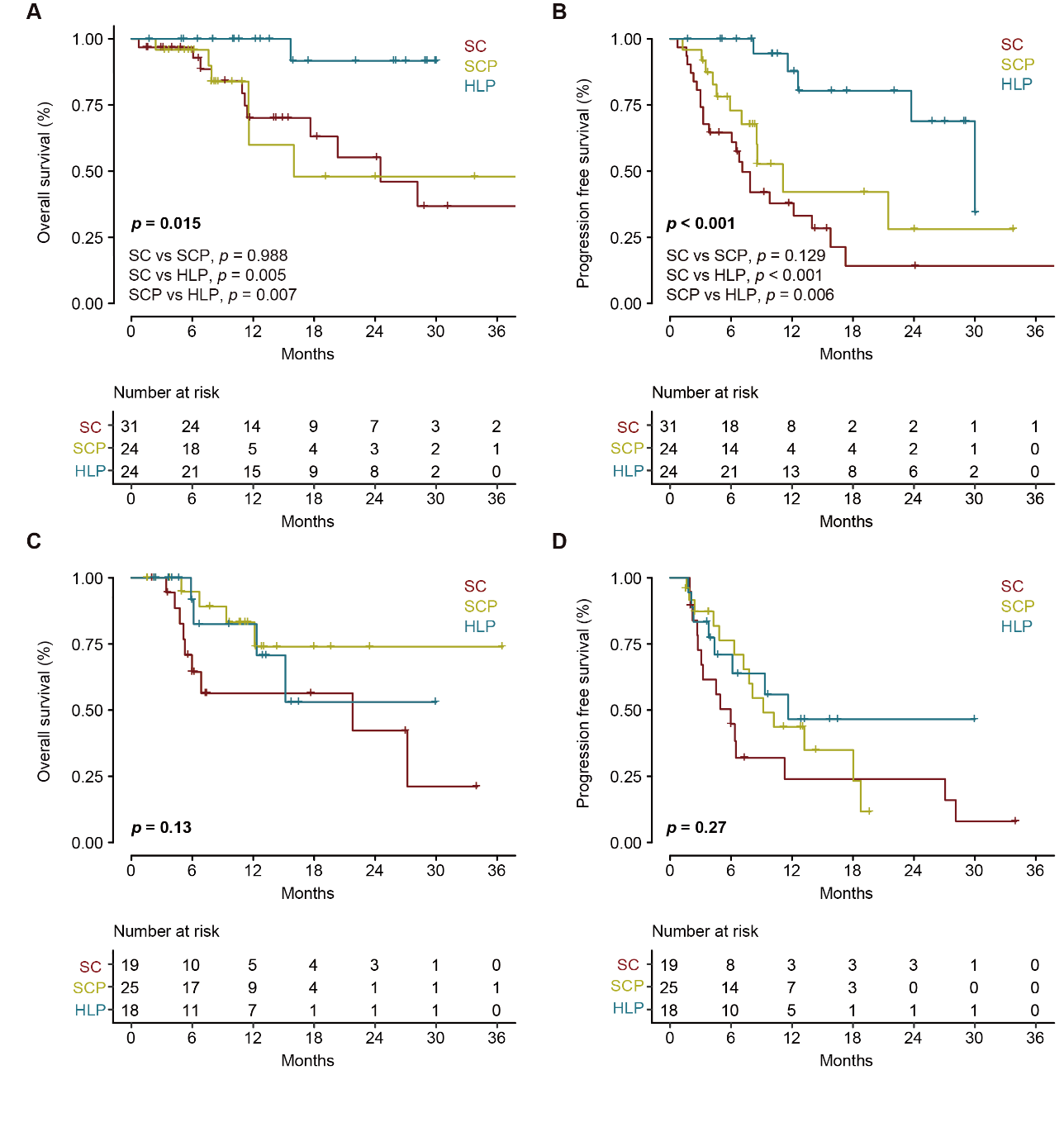
Supplementary figure **1.** Kaplan-Meier plots for overall survival (A) and progression-free survival (B) in patients without extrahepatic metastasis. Kaplan-Meier plots for overall survival (C) and progression-free survival (D) in patients with extrahepatic metastasis. Abbreviations: SC, systemic chemotherapy; SCP, systemic chemotherapy with PD-(L)1 inhibitors; HLP, hepatic artery infusion chemotherapy with lenvatinib and PD-(L)1 inhibitors.
